# Importance of serum albumin in machine learning-based prediction of cognitive function in the elderly using a basic blood test

**DOI:** 10.3389/fneur.2024.1362560

**Published:** 2024-07-24

**Authors:** Kenji Karako, Takeo Hata, Atsushi Inoue, Katsunori Oyama, Eiichiro Ueda, Kaoru Sakatani

**Affiliations:** ^1^Department of Human and Engineered Environmental Studies, Graduate School of Frontier Sciences, The University of Tokyo, Chiba, Japan; ^2^Department of Hospital Quality and Safety Management, Osaka Medical and Pharmaceutical University Hospital, Osaka, Japan; ^3^Department of Pharmacy, Osaka Medical and Pharmaceutical University Hospital, Osaka, Japan; ^4^Graduate School of Life Science and Systems Engineering, Kyushu Institute of Technology, Fukuoka, Japan; ^5^Department of Computer Science, College of Engineering, Nihon University, Tokyo, Japan; ^6^Institute of Gerontology, The University of Tokyo, Tokyo, Japan

**Keywords:** albumin, blood test, cognitive function, deep learning, machine learning, mild cognitive impairment

## Abstract

**Introduction:**

In this study, we investigated the correlation between serum albumin levels and cognitive function, and examined the impact of including serum albumin values in the input layer on the prediction accuracy when forecasting cognitive function using deep learning and other machine learning models.

**Methods:**

We analyzed the electronic health record data from Osaka Medical and Pharmaceutical University Hospital between 2014 and 2021. The study included patients who underwent cognitive function tests during this period; however, patients from whom blood test data was not obtained up to 30 days before the cognitive function tests and those with values due to measurement error in blood test results were excluded. The Mini-Mental State Examination (MMSE) was used as the cognitive function test, and albumin levels were examined as the explanatory variable. Furthermore, we estimated MMSE scores from blood test data using deep learning models (DLM), linear regression models, support vector machines (SVM), decision trees, random forests, extreme gradient boosting (XGBoost), and light gradient boosting machines (LightGBM).

**Results:**

Out of 5,017 patients who underwent cognitive function tests, 3,663 patients from whom blood test data had not been obtained recently and two patients with values due to measurement error were excluded. The final study population included 1,352 patients, with 114 patients (8.4%) aged below 65 and 1,238 patients (91.6%) aged 65 and above. In patients aged 65 and above, the age and male sex showed significant associations with MMSE scores of less than 24, while albumin and potassium levels showed negative associations with MMSE scores of less than 24. Comparing MMSE estimation performance, in those aged below 65, the mean squared error (MSE) of DLM was improved with the inclusion of albumin. Similarly, the MSE improved when using SVM, random forest and XGBoost. In those aged 65 and above, the MSE improved in all models.

**Discussion:**

Our study results indicated a positive correlation between serum albumin levels and cognitive function, suggesting a positive correlation between nutritional status and cognitive function in the elderly. Serum albumin levels were shown to be an important explanatory variable in the estimation of cognitive function for individuals aged 65 and above.

## 1 Introduction

Dementia stands as a predominant etiology of impairment among the elderly, afflicting approximately 50 million individuals globally (1). Given the precipitous aging of the world population, this figure is anticipated to surge exponentially, exceeding 150 million by the year 2050. Consequently, dementia emerges as a paramount challenge in 21st-century realms of medical practice, public health, and societal care (2). Cholinesterase inhibitors, namely donepezil, galantamine, and rivastigmine, along with the *N*-methyl-D-aspartate (NMDA) receptor antagonist memantine, have hitherto served as therapeutic agents for dementia. Additionally, monoclonal antibodies targeting amyloid-β, such as aducanumab (3) approved by the United States Food and Drug Administration (FDA) in June 2021 and lecanemab (4) in July 2023 based on the amyloid-β cascade hypothesis (5, 6), have entered the treatment landscape. However, these interventions are primarily palliative, aiming to retard symptom progression, lacking fundamental disease-modifying properties, and exhibiting circumscribed clinical efficacy. Currently, as there is no fundamental cure for dementia, it is vital to take appropriate measures from an early stage to halt its progression.

Mild cognitive impairment (MCI) represents a cohort for evaluating early therapeutic interventions in Alzheimer’s disease. This is because MCI occupies an intermediate stage between normal functioning and Alzheimer’s disease, conferring a higher risk of cognitive decline compared to cognitively healthy elderly individuals (7, 8). Given the diverse progression rates among MCI patients, and considering that not all progress to Alzheimer’s disease, there is a need for tools to discern those MCI patients who would derive the utmost benefit from intervention (9).

To test for cognitive function, various methods are used, including biomarkers (10), and cognitive function measurements such as the Mini-Mental State Examination (MMSE) (11) and Hasegawa Dementia Scale-Revised (HDS-R) (12). These tests require an interview with a physician and are not suitable for mass screening for cognitive impairment. They can create a barrier to early detection, especially since patients in the early stages — who have few self-recognized symptoms — find it tiresome to undergo these tests voluntarily.

Midlife hypertension, obesity, and hypercholesterolemia are recognized as risk factors for late-onset dementia, including Alzheimer’s disease (13). Given the current absence of efficacious treatments to halt the progression of dementia, modifiable factors such as dietary intake play an indispensable role in the prevention and understanding of its etiology. The results of the Finnish Geriatric Intervention Study to Prevent Cognitive Impairment and Disability (FINGER), a double-blind randomized controlled trial evaluating the preventive effects of lifestyle intervention on cognitive decline in elderly Finns, were reported in 2015 (14). In the FINGER study, 1,260 individuals aged 60–77 with slight cognitive impairment were randomly assigned to an intervention group (*n* = 631) and a control group (*n* = 629) for a duration of 2 years. The intervention group received a multidomain intervention (diet, exercise, cognitive training, vascular risk monitoring), while the control group received conventional health advice. The results demonstrated that improving lifestyle factors effectively suppressed cognitive decline. Furthermore, recent reports have highlighted the association between poor nutritional status (15), frailty (16), lower albumin level (17–19), and cognitive decline, emphasizing these as modifiable elements of interest. Considering these challenges, there is ongoing development of methods to estimate cognitive decline using more readily available indicators.

Recently, with the remarkable progress in machine learning, particularly deep learning, there has been a surge in research applying these technologies to new disease diagnoses and early detection in the medical field. Studies are presently being conducted using deep learning to estimate cognitive decline using easily obtainable indicators such as facial (20), vocal (21), and blood test data (22). In light of the evidence establishing lifestyle-related diseases as risk factors for cognitive decline, our previous studies (22) have proposed using deep learning to estimate a patient’s MMSE score at the time of a blood test, using age, sex, and basic blood test data (24 blood items) as explanatory variables. This study demonstrated that by using machine learning, it is possible to quantitatively estimate the risk of cognitive impairment as MMSE scores, by inputting age, sex, and basic blood test data. For practical application in real clinical scenarios, it is preferable to have readily accessible patient information and construct a more streamlined model with fewer inputs. Given that health checkup data including basic blood test data already exist for the majority of elderly patients, estimating cognitive impairment risk from these data could potentially reduce unnecessary costs associated with cognitive function-related assessments.

Delaying the onset of dementia by 1 year may potentially result in an 11% reduction in the prevalence of dementia by 2050, and a five-year delay could lead to a halving of the population living with dementia by the same year (23). Given the incurable nature of dementia, early detection of cognitive impairment is of paramount importance. Therefore, the identification of factors particularly crucial for prediction becomes imperative. The current study centers its focus on serum albumin levels as a parameter believed to reflect nutritional status, investigating the association between MCI and albumin. Additionally, it explores variations in cognitive function estimation performance when considering serum albumin levels compared to when they we excluded.

## 2 Methods

### 2.1 Data source

We used electronic health record (EHR) data spanning an eight-year period from 2014 to 2021 from Osaka Medical and Pharmaceutical University Hospital. This is a university hospital and also provides medical services as a general hospital. It has 903 beds and 31 clinical departments and is located in Takatsuki City, Osaka Prefecture. The medical area is the Hokusetsu region, which has a population of approximately 1.65 million.

### 2.2 Ethical consideration

This study was conducted in accordance with the Declaration of Helsinki and approved by the ethics committee of Osaka Medical and Pharmaceutical University (Approval ID: 2022–181). Since this is a retrospective observational study without intervention or invasion, the requirement for informed consent was waived.

### 2.3 Construction of the study subject

Patients who underwent cognitive function tests at Osaka Medical and Pharmaceutical University Hospital from 2014 to 2021 were included. We applied the following exclusion criteria: no blood test data within 30 days prior to cognitive function tests and values due to measurement error in blood test data.

### 2.4 Outcome variable

The outcome was the results of cognitive function tests. Cognitive tests included HDS-R or MMSE. HDS-R values were converted to MMSE values for analysis as outcome measures. The conversion model from HDS-R to MMSE values was generated using the light gradient boosting machine (LightGBM) method (24), in 139 patients who had HDS-R and MMSE measured on the same day. Details of the conversion process are described in Section 2.6.

### 2.5 Explanatory variables

The following blood test data was obtained from EHR data and used in the analysis as explanatory variables: age, sex, white blood cell (WBC), red blood cell (RBC), hemoglobin, hematocrit, mean corpuscular volume (MCV), mean corpuscular hemoglobin (MCH), mean corpuscular hemoglobin concentration (MCHC), platelets, total protein, albumin, albumin-globulin ratio, aspartate aminotransferase (AST), alanine aminotransferase (ALT), γ-glutamyl transpeptidase (γ-GTP), total cholesterol, triglyceride, blood urea nitrogen (BUN), creatinine, uric acid, blood glucose, sodium, potassium, and chloride. With the exception of age and sex, all of these explanatory variables used data within 30 days prior to cognitive function measurement.

### 2.6 Conversion from HDS-R to MMSE

**Figure 1:**
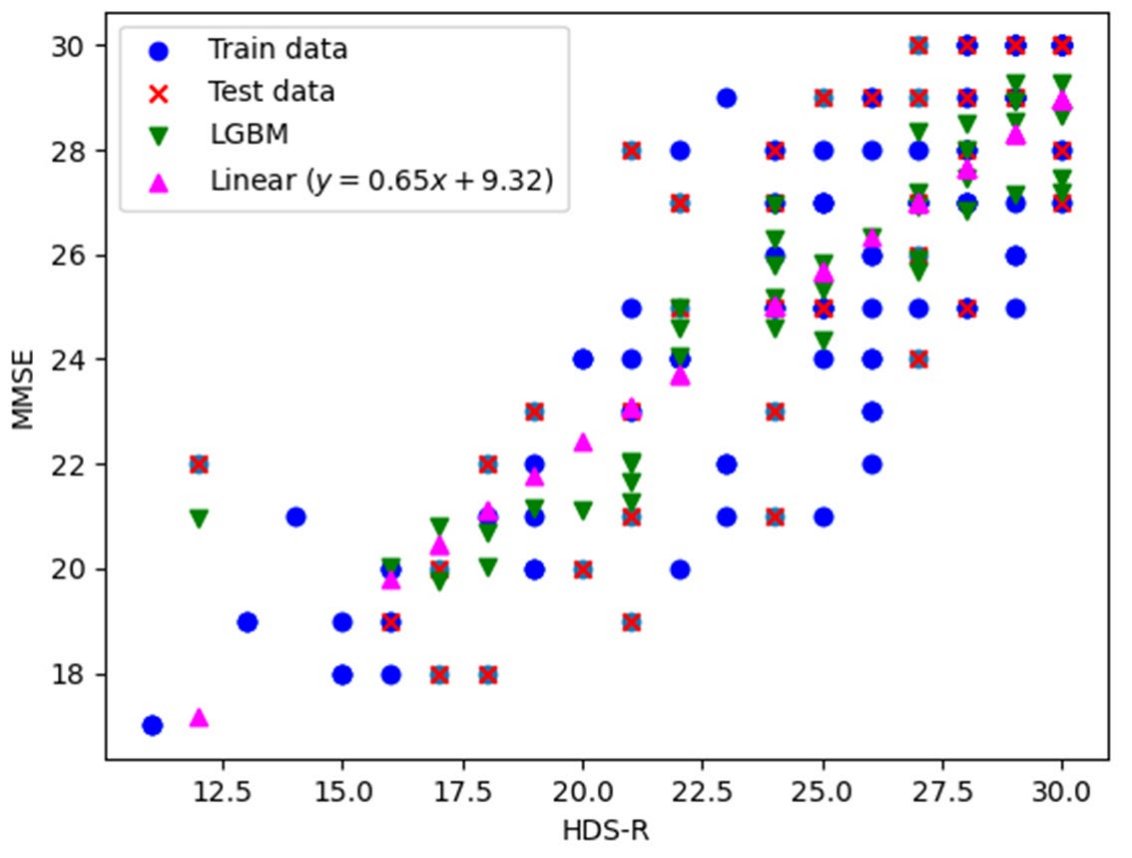
Training data, evaluation data, and prediction results plots for conversion from HDS-R to MMSE.

We explored the conversion from HDS-R to MMSE using both univariate linear regression and LightGBM to construct conversion models. Data from 139 patients revealed that the correspondence between HDS-R and MMSE was predominantly linear. Consequently, we constructed a univariate linear regression model predicting MMSE based solely on HDS-R and employed LightGBM with additional input variables of the individual’s blood test information described in Section 2.5. The data set of 139 patients was divided into a training set (97, 70%) and a test set (42, 30%), upon which the models were trained. The training data, test data, and the predicted MMSE-HDS-R relationship for each model with respect to the test data are shown in Figure 1. Results showed that the linear regression model (MMSE = 0.65 × HDS-R + 9.32) achieved mean squared error (MSE) of 5.245 and coefficient of determination (*R*^2^) of 0.630, while the LightGBM model recorded MSE of 4.555 and *R*^2^ of 0.679. Although the difference was slight, the superior outcomes demonstrated by LightGBM led to its selection for converting HDS-R to MMSE.

### 2.7 Statistical analysis

For comparison between the two groups, the Wilcoxon rank-sum test was applied to the numerical data and Fisher’s exact test to the categorical data. The adoption of the Wilcoxon rank-sum test for comparing numerical data in this study was due to preliminary analyses using the Kolmogorov–Smirnov test and QQ plots indicating that the data did not follow a normal distribution. It is believed that non-parametric tests offer more reliable results than parametric tests when the assumption of normality is not satisfied. The Wilcoxon test, which does not depend on the shape of the data distribution and compares two independent groups based on differences in medians, was deemed appropriate. The MMSE was divided into two categories as the objective variable, less than 24 or 24 or greater, and logistic regression analysis was used to examine the relationship between cognitive function and each test data. An MMSE score of less than 24 was used as a criterion for indicating MCI, in alignment with established benchmarks in the field of aging research. This threshold is supported by numerous studies (11, 25, 26) that validate the MMSE as a reliable tool for identifying individuals at an increased risk of dementia. Variables that were *p* < 0.05 by univariate logistic regression analysis were entered into the model as explanatory variables in the multivariate logistic regression analysis. This was to identify variables that were associated with MCI, as this approach has been previously used for similar analyses (27–29). Variance inflation factors (VIFs) ≥10 were considered evidence of multicollinearity. All *p*-values were reported using two-tailed tests and the significance level was set at 5%. Analyses were performed using R version 4.2.2 (R Development Core Team, Vienna, Austria).

### 2.8 Prediction algorithms for cognitive function

To investigate how each blood test item affects the prediction of cognitive function based on the above statistical analysis, we constructed predictive models to estimate the MMSE score using multiple common machine learning models and deep learning models (DLM). Similar to the statistical analysis, predictive models were built specifically for groups aged below 65 and those aged 65 and above. The explanatory variables used were those items that had been identified as having significant differences in the univariate logistic regression analysis described above. The algorithms used were linear regression model (LRM), support vector machine (SVM) (30), decision tree (31), random forest (32), extreme gradient boosting (XGBoost) (33), LightGBM (24), and DLM. The implementation of basic machine learning models such as LRM, SVM, decision tree and random forest was done using the scikit-learn library (34) in Python 3.9.13. For the advanced algorithms combining decision trees and ensemble learning, XGBoost, LightGBM, XGBoost (35) and LightGBM (36) libraries were utilized, respectively.

**Figure 2:**
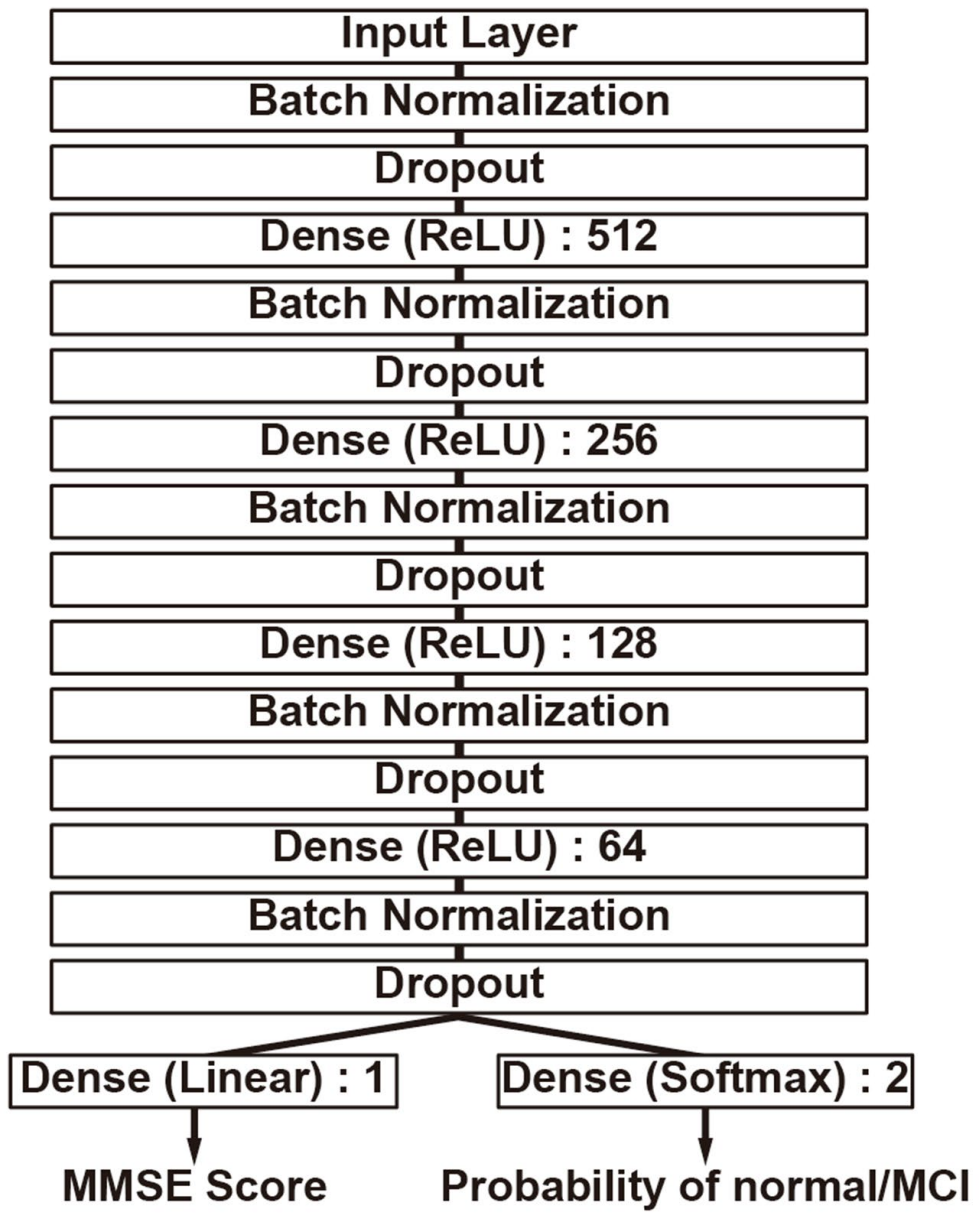
Deep learning model structure. It has two output layers, simultaneously performing MMSE score estimation and binary classification of the MMSE score as either less than 24 or 24 or greater. The numbers indicate the nodes of each neural network layer.

The construction of the DLM is based on a feedforward neural network (37) that consists of multiple fully connected layers, as shown in Figure 2. The fundamental architecture incorporates Dense layers (38) utilizing the ReLU (39) activation function, in addition to Batch normalization (40) and Dropout (41) mechanisms, combined into a unit and connected in four layers, with the final output layer splitting into two. Batch normalization stabilizes the variation in the distribution of input data during training, and Dropout is added to suppress overfitting and enhance the model’s robustness. Furthermore, one output layer is trained to estimate the MMSE score, while the other uses the Softmax activation function to estimate the probability that the input data is either normal or MCI. The MMSE score is an indicator for assessing cognitive function, where distinguishing between normal and MCI is particularly crucial. Thus, in anticipation of improving performance, an output layer for class classification has been added to take into account whether the condition is normal or MCI when estimating the MMSE score. During training, the weight ratio of the MMSE output value to the class classification output for the loss function is set as 1:0.001, prioritizing MMSE output, and only the MMSE output is used as the output of the DLM. Neural network construction was carried out using TensorFlow (42).

### 2.9 Data learning and evaluation

To predict MMSE using each model, the previously mentioned dataset was employed. To evaluate the performance of each model, three-fold cross-validation was conducted on the dataset. While five-fold or ten-fold cross-validation is commonly adopted, three-fold was chosen in this study to reduce computational costs due to the subsequent parameter optimization and the large number of models involved. The dataset was split into three groups, with each group serving as the evaluation data and the remaining two as training data. This process was conducted in three patterns, and the average of these evaluations was taken as the final assessment. Since the performance of each model obtained through training varied depending on the model’s hyperparameters, parameter optimization was conducted to evaluate the model’s performance using the best results obtained. For parameter optimization, a library called Optuna (43) was used. MSE was set as the evaluation function, and a hyperparameter search was conducted to minimize the MSE while varying the hyperparameters of each algorithm. This search was performed 100 times for each model, and the model that finally yielded the lowest MSE was evaluated. In addition to the MSE, we also used mean absolute error (MAE), root mean squared error (RMSE) and *R*^2^ for the evaluation of each model. In addition, we evaluate the performance of distinguishing whether a patient is normal or has MCI by calculating the Receiver Operating Characteristic and the Area Under the Curve (AUC) using the estimated MMSE scores from each model. The correct labels are treated as MCI if the actual MMSE score is below 24, and normal if it is 24 or above. The evaluation assesses the ability to differentiate based on the predicted MMSE scores.

## 3 Results

### 3.1 Study subjects

**Figure 3:**
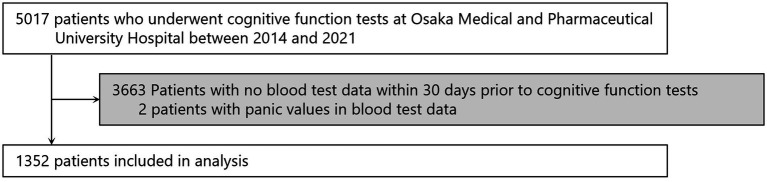
Screening of the study population. Some patients entered multiple times, so the numbers represent the total number of patients.

The flow for selecting participants for the study is shown in Figure 3. From the 5,017 patients who underwent cognitive function testing at Osaka Medical and Pharmaceutical University Hospital from 2014 to 2021, we excluded 3,663 patients who had not had blood tests performed within 30 days prior to the date of cognitive function testing and two patients whose blood test data showed erroneous values, resulting in 1,352 patients for the study. Erroneous values were observed in two individuals, with RBC at 0.01 × 10^6^/μL and albumin at 0.8 g/dL.

### 3.2 Demographic and clinical characteristics of the patients

Table 1 shows the background of the study patients. Of the 1,352 total, 114 (8.4%) were aged below 65 years and 1,238 (91.6%) were aged 65 and above. MMSE was significantly lower in those aged 65 and above, and the proportion of MMSE<24 with suspected MCI was 18.4% in those aged below 65 and 51.1% in those aged 65 years and above. Values of RBC, hemoglobin, hematocrit, platelets, albumin, albumin-globulin ratio, ALT, γ-GTP and triglyceride were significantly lower, and values of MCV, MCH, BUN, creatinine, uric acid, glucose, and potassium were significantly higher in those aged 65 and above. It is widely acknowledged that individuals aged 65 and over are classified as elderly according to the definition by the World Health Organization (WHO). Indeed, many previous studies focusing on dementia have employed 65 years as the cutoff value (15, 17–19). Supplementary Table S1 summarizes the background of 3,665 patients who were excluded. The distribution of MMSE scores, age, and gender was similar to that of the 1,352 subjects included in the study. Supplementary Table S2 presents the patient background categorized by MMSE scores. Comparing patients with MMSE scores below 24 and those with scores of 24 or higher, the mean ages were 79 and 75 years, respectively. The percentage of males was 51.5 and 42.8%. Hemoglobin levels were 12.4 g/dL and 12.8 g/dL, albumin levels were 3.7 g/dL and 4.0 g/dL, and creatinine levels were 0.84 mg/dL and 0.79 mg/dL, respectively.

**Table 1:** Background of the patients.

Backgrounds	Overall	<65 years old	≥65 years old	*p*-value
Patients, *n* (%)	1,352 (100)	114 (8.4)	1,238 (91.6)	
MMSE, median (IQR)	24 (22–27)	28 (26–29)	23 (22–27)	<0.001^*^
MMSE, *n* (%)				<0.001^*^
<24	653 (48.3)	21 (18.4)	632 (51.1)	
≥24	699 (51.7)	93 (81.6)	606 (48.9)	
Age, year, median (IQR)	77 (72–82)	56 (49–62)	78 (74–82)	<0.001^*^
Sex, *n* (%)				0.695
Female	717 (53.0)	58 (50.9)	659 (53.2)	
Male	635 (47.0)	56 (49.1)	579 (46.8)	
WBC, 10^3^/μL, median (IQR)	5.78 (4.77–7.11)	5.97 (4.62–7.32)	5.77 (4.78–7.09)	0.731
RBC, 10^6^/μL, median (IQR)	4.06 (3.68–4.42)	4.36 (3.86–4.59)	4.04 (3.67–4.39)	<0.001^*^
Hemoglobin, g/dL, median (IQR)	12.7 (11.5–13.7)	13.4 (11.9–14.1)	12.6 (11.4–13.6)	0.002^*^
Hematocrit, %, median (IQR)	38.2 (34.9–41.2)	40.3 (36.3–42.9)	38.2 (34.7–41.1)	0.002^*^
MCV, fL, median (IQR)	94.2 (90.8–98.1)	92.4 (88.4–96.7)	94.3 (91.0–98.1)	0.001^*^
MCH, pg., median (IQR)	31.2 (30.1–32.4)	30.8 (29.6–31.8)	31.2 (30.1–32.5)	0.009^*^
MCHC, %, median (IQR)	33.0 (32.2–33.7)	33.0 (32.2–33.9)	33.0 (32.3–33.7)	0.986
Platelets, 10^3^/μL, median (IQR)	210 (170–256)	237 (200–297)	208 (168–254)	<0.001^*^
Total protein, g/dL, median (IQR)	6.8 (6.4–7.2)	6.8 (6.4–7.2)	6.8 (6.4–7.2)	0.909
Albumin, g/dL, median (IQR)	3.9 (3.5–4.2)	4.0 (3.5–4.3)	3.9 (3.5–4.1)	0.012^*^
Albumin-globulin ratio, median (IQR)	1.3 (1.1–1.5)	1.4 (1.1–1.6)	1.3 (1.1–1.5)	0.001^*^
AST, U/L, median (IQR)	21 (17–26)	21 (16–31)	21 (17–26)	0.950
ALT, U/L, median (IQR)	16 (11–23)	20 (14–30)	15 (11–22)	<0.001^*^
γ-GTP, U/L, median (IQR)	23 (16–39)	29 (18–61)	23 (16–38)	0.001^*^
Total cholesterol, mg/dL, median (IQR)	188 (162–216)	198 (163–225)	188 (162–216)	0.189
Triglyceride, mg/dL, median (IQR)	105 (76–146)	114 (86–173)	104 (75–143)	0.002^*^
BUN, mg/dL, median (IQR)	17 (13–21)	14 (10–18)	17 (14–22)	<0.001^*^
Creatinine, mg/dL, median (IQR)	0.81 (0.66–1.01)	0.72 (0.60–0.82)	0.83 (0.67–1.04)	<0.001^*^
Uric acid, mg/dL, median (IQR)	4.9 (4.0–6.0)	4.6 (3.8–5.3)	4.9 (4.0–6.0)	0.004^*^
Glucose, mg/dL, median (IQR)	109 (94–147)	105 (88–128)	111 (95–148)	<0.001^*^
Sodium, mEq/L, median (IQR)	141 (139–143)	141 (139–143)	141 (139–143)	0.552
Potassium, mEq/L, median (IQR)	4.2 (3.9–4.5)	4.1 (3.8–4.3)	4.2 (3.9–4.5)	0.023^*^
Chloride, mEq/L, median (IQR)	104 (102–106)	104 (102–106)	104 (102–106)	0.910

ALT: alanine aminotransferase, AST: aspartate aminotransferase, BUN: blood urea nitrogen, γ-GTP: γ-glutamyl transpeptidase, IQR: interquartile range, MCH: mean corpuscular hemoglobin, MCHC: mean corpuscular hemoglobin concentration, MCV: mean corpuscular volume, MMSE: Mini Mental State Examination, RBC: red blood cell, WBC: white blood cell.

^*^p < 0.05.

### 3.3 Relationship between cognitive function and blood test data

For each blood test data, the association with MMSE<24 was examined using logistic regression analysis. Multivariate logistic regression analysis was performed using a model that included platelets, total protein, albumin, triglyceride, uric acid, and sodium, which was *p* < 0.05 by univariate logistic regression analysis in patients aged below 65. The results showed an association between platelets and MMSE<24 (Table 2). Conversely, in patients aged 65 and above, multivariate logistic regression analysis with WBC, RBC, total protein, albumin, BUN, creatinine, and potassium as explanatory variables, which were *p* < 0.05 by univariate logistic regression analysis, showed an association between MMSE<24 and age or male, and a negative association between MMSE<24 and albumin or potassium. Hemoglobin, hematocrit, and albumin-globulin ratio were not included as explanatory variables due to multicollinearity (Table 3).

**Table 2:** Relationship between blood test data and MMSE<24 in patients under 65 years of age.

	Unadjusted		Adjusted		
Variables	OR (95%CI)	*p*-value	OR (95%CI)	*p*-value	VIF
Age, years, (per 1 unit)	1.01 (0.96–1.06)	0.832	0.99 (0.94–1.05)	0.830	1.08
Sex, (male)	1.17 (0.46–3.03)	0.741	1.89 (0.60–6.37)	0.283	1.20
WBC, 10^3^/μL, (per 1 unit)	1.20 (0.99–1.46)	0.069			
RBC, 10^6^/μL, (per 1 unit)	0.53 (0.25–1.11)	0.089			
Hemoglobin, g/dL, (per 1 unit)	0.85 (0.67–1.07)	0.159			
Hematocrit, %, (per 1 unit)	0.94 (0.87–1.03)	0.186			
MCV, fL, (per 1 unit)	1.06 (0.99–1.12)	0.089			
MCH, pg., (per 1 unit)	1.10 (0.91–1.34)	0.311			
MCHC, %, (per 1 unit)	0.80 (0.55–1.16)	0.239			
Platelets, 10^3^/μL, (per 1 unit)	1.01 (1.00–1.01)	0.003^*^	1.01 (1.00–1.01)	0.041^*^	1.45
Total protein, g/dL, (per 1 unit)	0.48 (0.24–0.95)	0.030^*^	0.49 (0.16–1.39)	0.197	1.70
Albumin, g/dL, (per 1 unit)	0.41 (0.20–0.84)	0.015^*^	1.38 (0.38–5.30)	0.630	2.30
Albumin-globulin ratio, (per 1 unit)	0.38 (0.10–1.52)	0.171			
AST, U/L, (per 1 unit)	1.00 (0.99–1.01)	0.728			
ALT, U/L, (per 1 unit)	1.00 (0.99–1.02)	0.717			
γ-GTP, U/L, (per 1 unit)	1.01 (1.00–1.02)	0.062			
Total cholesterol, mg/dL, (per 1 unit)	0.99 (0.98–1.00)	0.096			
Triglyceride, mg/dL, (per 1 unit)	0.99 (0.98–1.00)	0.022^*^	0.99 (0.98–1.00)	0.216	1.22
BUN, mg/dL, (per 1 unit)	1.00 (0.94–1.07)	0.898			
Creatinine, mg/dL, (per 1 unit)	0.38 (0.05–2.79)	0.294			
Uric acid, mg/dL, (per 1 unit)	0.59 (0.40–0.85)	0.003^*^	0.72 (0.45–1.11)	0.153	1.35
Glucose, mg/dL, (per 1 unit)	1.00 (0.99–1.01)	0.430			
Sodium, mEq/L, (per 1 unit)	0.87 (0.76–0.99)	0.035^*^	0.94 (0.79–1.12)	0.510	1.31
Potassium, mEq/L, (per 1 unit)	1.07 (0.33–3.49)	0.911			
Chloride, mEq/L, (per 1 unit)	1.01 (0.88–1.16)	0.888			

ALT: alanine aminotransferase, AST: aspartate aminotransferase, BUN: blood urea nitrogen, CI: confidence interval, γ-GTP: γ-glutamyl transpeptidase, MCH: mean corpuscular hemoglobin, MCHC: mean corpuscular hemoglobin concentration, MCV: mean corpuscular volume, MMSE: Mini Mental State Examination, OR: odds ratio, RBC: red blood cell, VIF: variance inflation factor, WBC: white blood cell.

^*^p < 0.05.

**Table 3:** Relationship between blood test data and MMSE<24 in patients 65 years and older.

	Unadjusted		Adjusted		
Variables	OR (95%CI)	*p*-value	OR (95%CI)	*p*-value	VIF
Age, years, (per 1 unit)	1.07 (1.05–1.09)	<0.001^*^	1.06 (1.04–1.09)	<0.001^*^	1.06
Sex, (male)	1.47 (1.17–1.84)	<0.001^*^	1.44 (1.13–1.84)	0.003^*^	1.07
WBC, 10^3^/μL, (per 1 unit)	1.06 (1.01–1.12)	0.011*	1.05 (1.00–1.12)	0.070	1.12
RBC, 10^6^/μL, (per 1 unit)	0.62 (0.50–0.75)	<0.001^*^	0.84 (0.65–1.08)	0.175	1.50
Hemoglobin, g/dL, (per 1 unit)	0.85 (0.80–0.91)	<0.001^*^			
Hematocrit, %, (per 1 unit)	0.95 (0.93–0.97)	<0.001^*^			
MCV, fL, (per 1 unit)	1.02 (1.00–1.04)	0.075			
MCH, pg., (per 1 unit)	1.01 (0.96–1.06)	0.758			
MCHC, %, (per 1 unit)	0.88 (0.80–0.97)	0.010^*^			
Platelets, 10^3^/μL, (per 1 unit)	1.00 (1.00–1.00)	0.696			
Total protein, g/dL, (per 1 unit)	0.68 (0.57–0.81)	<0.001^*^	1.04 (0.82–1.33)	0.739	1.77
Albumin, g/dL, (per 1 unit)	0.42 (0.34–0.52)	<0.001^*^	0.53 (0.38–0.73)	<0.001^*^	2.05
Albumin-globulin ratio, (per 1 unit)	0.24 (0.16–0.36)	<0.001^*^			
AST, U/L, (per 1 unit)	1.00 (1.00–1.01)	0.458			
ALT, U/L, (per 1 unit)	1.00 (0.99–1.01)	0.869			
γ-GTP, U/L, (per 1 unit)	1.00 (1.00–1.00)	0.116			
Total cholesterol, mg/dL, (per 1 unit)	1.00 (1.00–1.00)	0.096			
Triglyceride, mg/dL, (per 1 unit)	1.00 (1.00–1.00)	0.607			
BUN, mg/dL, (per 1 unit)	1.02 (1.00–1.03)	0.005^*^	0.99 (0.98–1.01)	0.511	2.01
Creatinine, mg/dL, (per 1 unit)	1.27 (1.11–1.46)	<0.001^*^	1.16 (0.98–1.40)	0.099	1.86
Uric acid, mg/dL, (per 1 unit)	1.02 (0.95–1.09)	0.597			
Glucose, mg/dL, (per 1 unit)	1.00 (1.00–1.00)	0.912			
Sodium, mEq/L, (per 1 unit)	0.98 (0.95–1.01)	0.227			
Potassium, mEq/L, (per 1 unit)	0.77 (0.62–0.97)	0.023^*^	0.77 (0.59–0.99)	0.040^*^	1.16
Chloride, mEq/L, (per 1 unit)	1.00 (0.97–1.03)	0.973			

ALT: alanine aminotransferase, AST: aspartate aminotransferase, BUN: blood urea nitrogen, CI: confidence interval, γ-GTP: γ-glutamyl transpeptidase, MCH: mean corpuscular hemoglobin, MCHC: mean corpuscular hemoglobin concentration, MCV: mean corpuscular volume, MMSE: Mini Mental State Examination, OR: odds ratio, RBC: red blood cell, VIF: variance inflation factor, WBC: white blood cell.

^*^p < 0.05.

### 3.4 Evaluation of cognitive function prediction model performance

**Figure 4:**
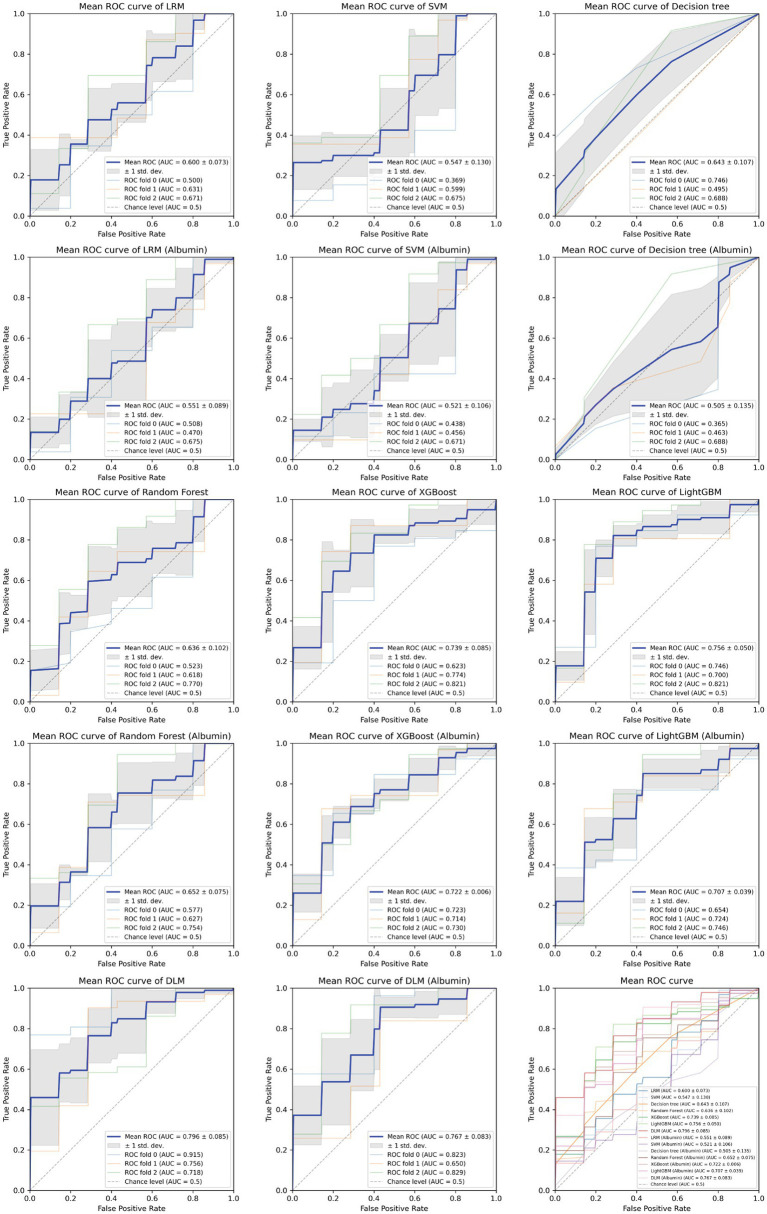
Receiver operating characteristic curve for each MMSE regression model in under 65 years old.

**Figure 5:**
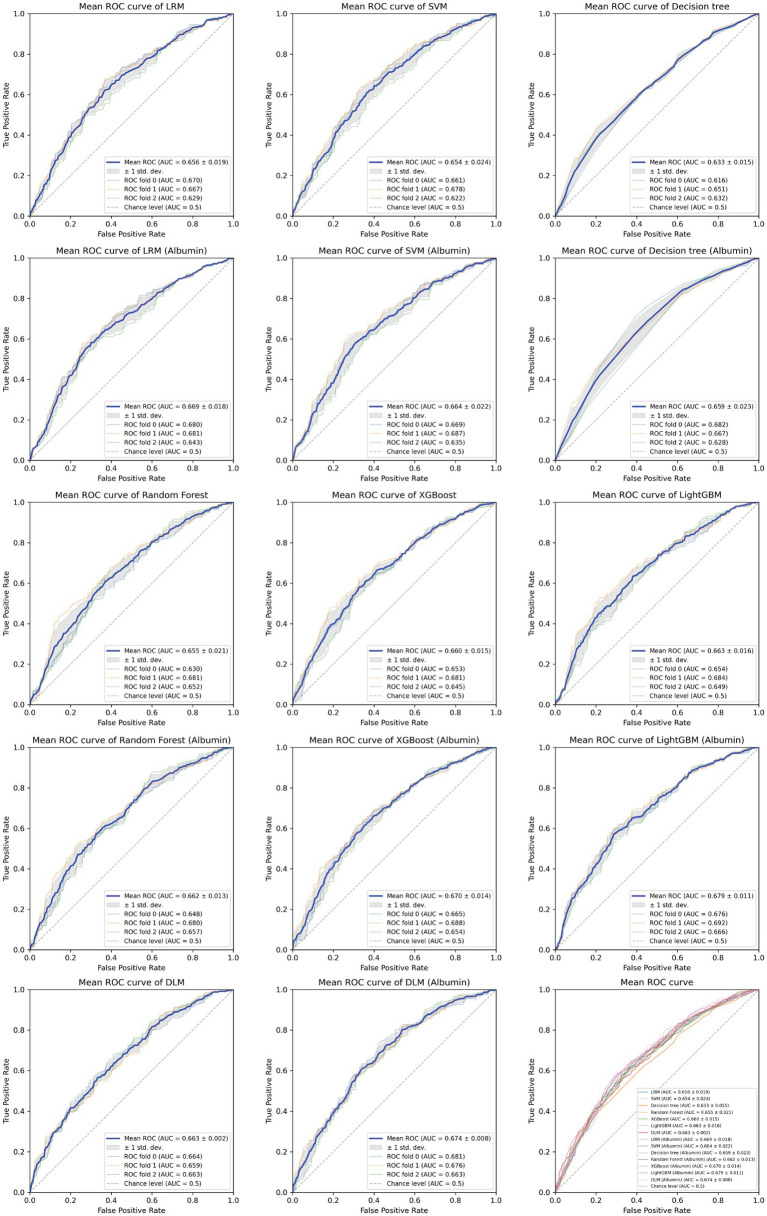
Receiver operating characteristic curve for each MMSE regression model in those aged 65 and above.

**Figure 6:**
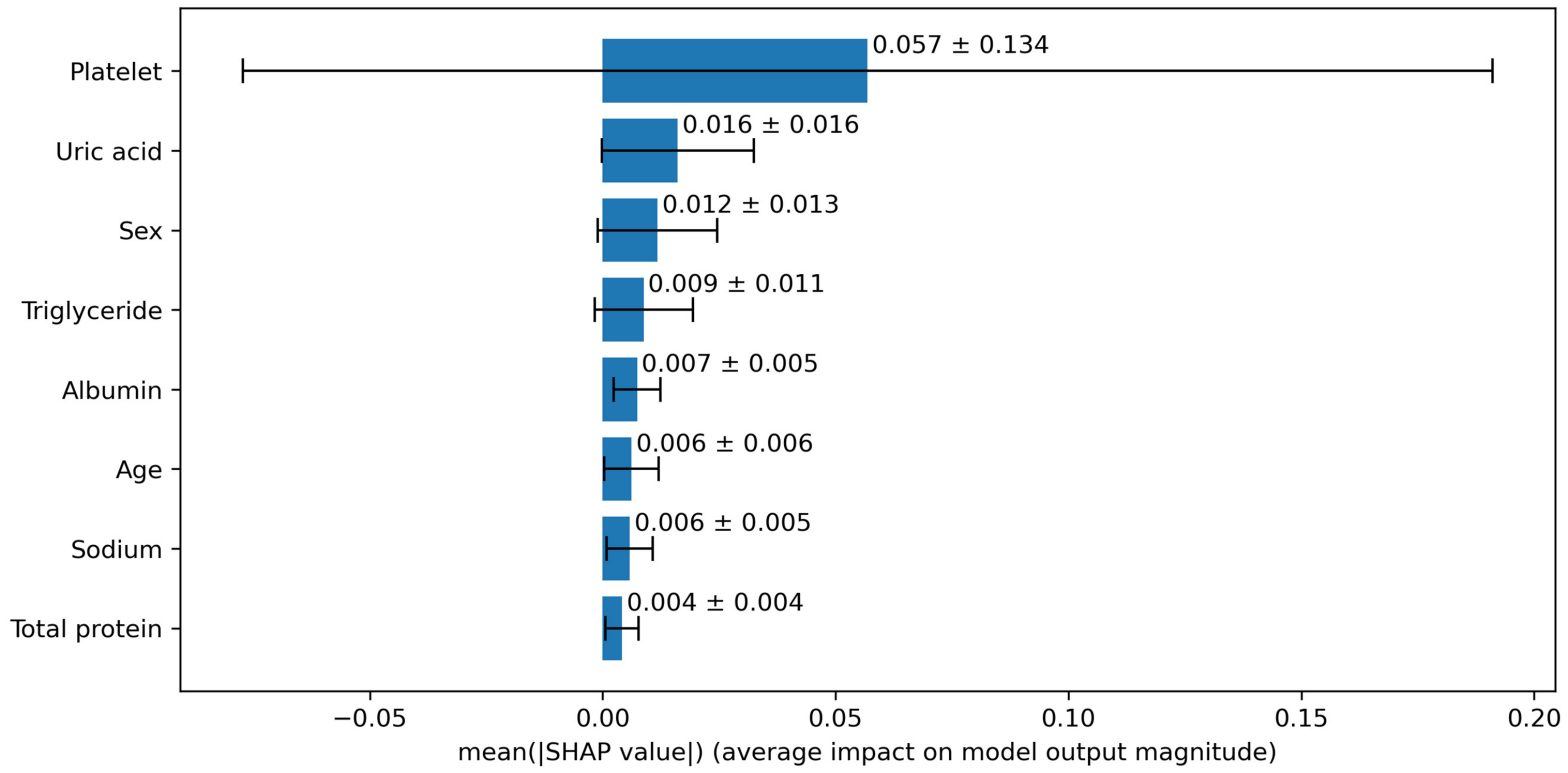
Impact of SHAP value for deep learning model in under 65 years old.

**Figure 7:**
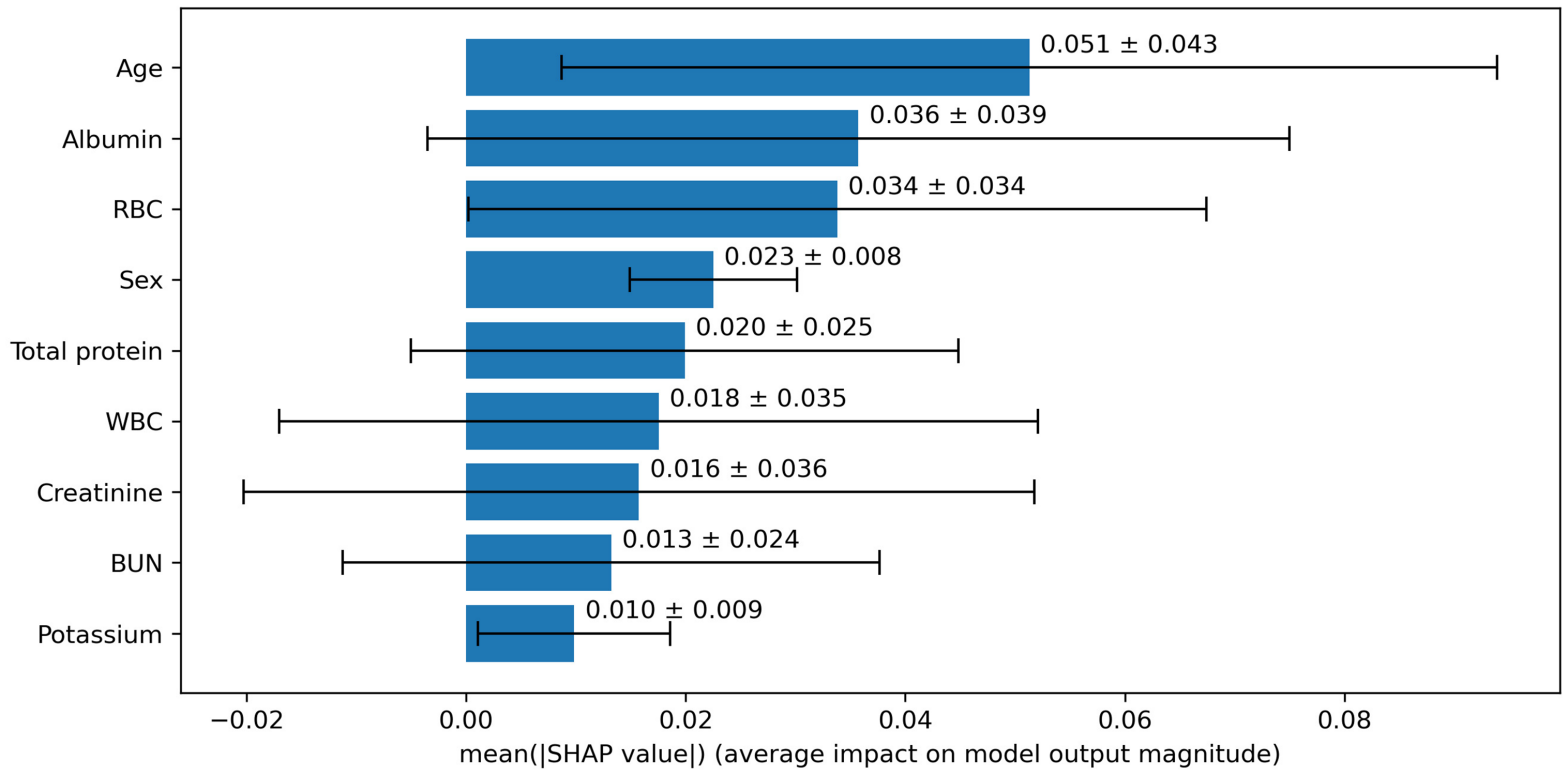
Impact of SHAP value for deep learning model in those aged 65 and above.

Based on the aforementioned statistical analysis, which indicated that the variables with a significant impact differ between the patients aged below 65 and the patients aged 65 and above, the dataset was divided into two age groups: aged below 65 and aged 65 and above. Models for estimating MMSE scores were then constructed and evaluated for each age group using three-fold cross-validation. The variables listed in Table 4, which showed significant differences through statistical analysis for each age group, were adopted as explanatory variables. Furthermore, to compare the estimation performance when including albumin or not, models were also constructed excluding albumin as an explanatory variable. The results of the three-fold cross-validation for these models are presented in Table 5. Figures 4, 5 show the ROC plots evaluating each model constructed for groups aged below 65 and aged 65 and above, respectively, as a binary classification problem of normal or MCI. The best results for both aged below 65 and aged 65 and above datasets were obtained using the DLM and including albumin. For the aged below 65 dataset, comparing performance when including albumin or not, the DLM showed an improvement from an MSE of 6.325 to 5.357, a reduction of −0.968 with the inclusion of albumin. In contrast, no improvement was observed with the LRM, decision tree, random forest, XGBoost, or LightGBM. In the aged 65 and above group, the MSE of the DLM was 6.370 when including albumin and 6.431 when not, showing a − 0.061 improvement with the inclusion of albumin. When using other typical machine learning models, the MSE improved in all models, including LRM, SVM, decision trees, random forest, XGBoost, and LightGBM. When comparing the R^2^, which indicates how well the model fits the data, between models with and without albumin, for the group aged below 65, the use of albumin in SVM models improved from −0.121 to −0.098, a 0.023 improvement, and in DLM models from 0.035 to 0.182, a 0.147 improvement. For other models, performance worsened. In the group aged 65 and above, all models showed improvement when albumin was used, with an average improvement of 0.013 ± 0.004. Although the improvement margin is small, it confirms that albumin contributes to the estimation of MMSE scores in the group aged 65 and over. When comparing the results of the AUC for the group aged below 65, only the model using albumin in the random forest showed improvement, from 0.636 to 0.652, an improvement of 0.015, while other models worsened when albumin was used. For the group aged 65 and above, all models improved when albumin was used, with an average improvement of 0.013 ± 0.006. This, similar to the R^2^ results, confirms that albumin contributes to the determination of normal versus MCI status using MMSE score estimations. Figures 6, 7 display SHapley Additive exPlanations (SHAP) (44) values of the DLM, which exhibited the most superior performance in aged below 65 and aged 65 and above groups. SHAP values quantify the extent to which each input feature contributes to the predicted output. Widespread SHAP values indicate a substantial impact on the prediction of cognitive function. In the group aged 65 or above, albumin emerged as a crucial variable in the cognitive function prediction model, following age.

**Table 4:** Explanatory variables for the two age groups.

<65 years old	≥65 years old
Age, years	Age, years
Sex, (male)	Sex, (male)
Platelets, 10^3^/μL	WBC, 10^3^/μL
Total protein, g/dL	RBC, 10^6^/μL
Albumin, g/dL	Total protein, g/dL
Triglyceride, mg/dL	Albumin, g/dL
Uric acid, mg/dL	BUN, mg/dL
Sodium, mEq/L	Creatinine, mg/dL
	Potassium, mEq/L

BUN: blood urea nitrogen, RBC: red blood cell, WBC: white blood cell.

**Table 5:** Performance of MMSE regression model.

Used Model	Used Albumin	<65 years old						≥65 years old					
		corr	MAE	MSE	RMSE	*R* ^2^	AUC	corr	MAE	MSE	RMSE	*R* ^2^	AUC
LRM	No	0.178	2.164	7.031	2.651	−0.085	0.600	0.333	2.263	6.663	2.581	0.101	0.656
		(0.102)	(0.166)	(0.359)	(0.067)	(0.134)	(0.073)	(0.032)	(0.026)	(0.172)	(0.033)	(0.017)	(0.019)
SVM	No	0.225	2.042	7.272	2.693	−0.121	0.547	0.329	2.226	7.074	2.659	0.046	0.654
		(0.129)	(0.117)	(0.787)	(0.149)	(0.164)	(0.130)	(0.029)	(0.031)	(0.310)	(0.058)	(0.024)	(0.024)
Decision tree	No	0.265	1.962	6.362	2.517	0.026	0.643	0.300	2.287	6.875	2.622	0.072	0.633
		(0.143)	(0.122)	(0.864)	(0.168)	(0.115)	(0.107)	(12.000)	(0.010)	(0.222)	(0.043)	(0.014)	(0.015)
Random forest	No	0.240	1.998	6.207	2.485	0.041	0.636	0.343	2.268	6.619	2.572	0.107	0.655
		(0.248)	(0.165)	(0.834)	(0.172)	(0.171)	(0.102)	(0.008)	(0.026)	(0.169)	(0.033)	(0.008)	(0.021)
XGBoost	No	0.338	1.985	5.895	2.428	0.093	0.739	0.355	2.259	6.521	2.553	0.120	0.660
		(0.141)	(0.044)	(0.179)	(0.037)	(0.080)	(0.085)	(0.022)	(0.028)	(0.223)	(0.044)	(0.014)	(0.015)
LightGBM	No	0.332	1.986	5.862	2.419	0.099	0.756	0.351	2.260	6.560	2.561	0.115	0.663
		(0.150)	(0.070)	(0.533)	(0.110)	(0.098)	(0.050)	(0.017)	(0.028)	(0.206)	(0.040)	(0.011)	(0.016)
DLM	No	0.376	2.123	6.325	2.509	0.035	0.796	0.367	2.215	6.431	2.536	0.132	0.663
		(0.098)	(0.157)	(0.903)	(0.178)	(0.091)	(0.085)	(0.011)	(0.035)	(0.119)	(0.023)	(0.009)	(0.002)
LRM	Yes	0.126	2.196	7.655	2.764	−0.177	0.551	0.350	2.245	6.581	2.565	0.112	0.669
		(0.071)	(0.130)	(0.679)	(0.123)	(0.124)	(0.089)	(0.035)	(0.032)	(0.171)	(0.033)	(0.021)	(0.018)
SVM	Yes	0.199	1.987	7.146	2.669	−0.098	0.521	0.343	2.216	7.003	2.646	0.055	0.664
		(0.102)	(0.105)	(0.827)	(0.155)	(0.138)	(0.106)	(0.024)	(0.026)	(0.210)	(0.040)	(0.012)	(0.022)
Decision tree	Yes	0.169	2.002	6.848	2.610	−0.054	0.505	0.323	2.274	6.718	2.592	0.093	0.659
		(0.197)	(0.140)	(0.943)	(0.183)	(0.164)	(0.135)	(0.015)	(0.030)	(0.116)	(0.022)	(0.005)	(0.023)
Random forest	Yes	0.237	1.983	6.287	2.503	0.031	0.652	0.359	2.251	6.536	2.557	0.118	0.662
		(0.221)	(0.159)	(0.738)	(0.150)	(0.149)	(0.075)	(0.004)	(0.021)	(0.115)	(0.023)	(0.003)	(0.013)
XGBoost	Yes	0.334	1.995	5.895	2.428	0.093	0.722	0.378	2.232	6.419	2.533	0.134	0.670
		(0.113)	(0.058)	(0.212)	(0.044)	(0.080)	(0.006)	(0.014)	(0.048)	(0.206)	(0.041)	(0.012)	(0.014)
LightGBM	Yes	0.316	2.012	6.149	2.477	0.055	0.707	0.377	2.240	6.435	2.536	0.131	0.679
		(0.133)	(0.080)	(0.552)	(0.111)	(0.099)	(0.039)	(0.018)	(0.026)	(0.160)	(0.032)	(0.009)	(0.011)
DLM	Yes	0.471	1.787	5.357	2.303	0.182	0.767	0.380	2.208	6.370	2.524	0.140	0.674
		(0.114)	(0.111)	(1.089)	(0.228)	(0.140)	(0.083)	(0.024)	(0.034)	(0.102)	(0.020)	(0.014)	(0.008)

AUC: Area under the receiver operating characteristic curve, Corr.: Pearson product–moment correlation coefficient, DLM: deep learning model, LRM: linear regression model, MAE: mean absolute error, MSE: mean squared error, RMSE: root mean squared error, R^2^: Coefficient of determination, SVM: support vector machine.

## 4 Discussion

We obtained EHR data from blood tests that are easily accessible and conducted in the most extensive cohort among various examinations, investigating the association between MCI and albumin. From the results of the statistical analysis, our study indicated a correlation between low serum albumin levels and decreased cognitive function in the group aged 65 and older. We investigated alterations in performance of machine learning-based assessment of cognitive impairment when including and excluding serum albumin levels, a parameter indicative of nutritional status. The inclusion of albumin as an input was observed to improve performance in patients aged 65 and above, while in those aged below 65, improvement was observed only in some models. According to SHAP values, which calculate the importance of each variable on the prediction outcomes, albumin was the second most impactful variable in the group aged 65 and above. In the aged below 65 group, albumin ranked fifth, indicating that it was not as significant in this group. These findings align with the results of statistical analysis. It becomes evident that albumin is a crucial indicator in evaluating cognitive function in individuals aged 65 and above.

In this study, an association between low albumin and MCI was observed in the group aged 65 and above. While the fundamental mechanism underlying the correlation between serum albumin and cognitive function remains unidentified, insights from a limited clinical sample suggest a potential association between decreased serum albumin concentration and cognitive impairment. Numerous reports indicate that low albumin levels are correlated with diminished cognitive function. A retrospective cohort study involving a total of 2,396 Korean military veterans and their families aged 65 and above suggested that a sustained decline in serum albumin levels is associated with a decrease in MMSE scores (18). Cross-sectional studies utilizing clinical samples, including 1,827 community-dwelling elderly Japanese individuals (45), 1,511 hospitalized heart failure patients (46), 331 rehabilitation patients with hip fractures (47), and elderly patients with Alzheimer’s disease (48), demonstrated a correlation between decreased serum albumin levels and cognitive impairment. A nationally representative population-based study, involving 1,752 adults aged 65 and above who participated in the Health Survey for England 2000, revealed that low serum albumin levels were independently associated with an increased probability of cognitive impairment (19). In a study of 2,550 elderly individuals residing in Chinese communities, low serum albumin levels were independently associated with a decline in cognitive abilities (49). Subsequent research confirmed these cross-sectional findings, indicating a more significant cognitive decline over a 2-year follow-up period in older individuals with low serum albumin levels (50). In a study of 1,744 community-dwelling adults aged 65 and above participating in annual health check-ups in Japan, participants with the lowest baseline albumin levels (below the first quartile line) exhibited a significantly accelerated decline in MMSE scores over a 13-year period compared to those with the highest levels (above the third quartile line) (17). In a study involving 101 Alzheimer’s disease (AD) patients and 101 healthy controls, the AD group exhibited significant decreases in albumin, bilirubin, and uric acid levels (51). Our study’s result, indicating an association between low albumin and MCI in patients aged 65 and above, substantiates these previous research findings.

There are several reports on the relationship between nutritional status and cognitive impairment. Nutrition serves as a pivotal indicator for brain health and cognitive function (52). Multiple brain processes supporting cognitive function are contingent upon nutritional status, wherein nutrition plays a role in regulating neurotransmitter pathways, synaptic transmission, membrane fluidity, and signal transduction pathways (52). Inadequate protein intake, particularly in the elderly, may elevate the risk of sarcopenia and frailty, strongly correlating with the onset of cognitive impairment (53). Peptides rich in proline demonstrate a preventive effect on the progression of dementia (54); hence, consideration of protein supplementation is imperative for the elderly to delay cognitive decline. Furthermore, evidence suggests the involvement of inflammatory mechanisms in the pathogenesis of cognitive impairment and dementia (55, 56). Several epidemiological studies consistently demonstrate a significant association between systemic inflammatory markers, namely C-reactive protein (CRP) and tumor necrosis factor-α (TNF-α), and cognitive impairment or dementia. For instance, the increase in TNF-α associated with acute and chronic systemic inflammation is linked to the enhanced cognitive decline in Alzheimer’s disease (57). CRP may serve as a marker for memory impairment and visuospatial dysfunction in the elderly (58, 59). These findings support the notion that brain atrophy and cognitive decline in Alzheimer’s disease may be induced by acute and chronic systemic inflammation. Albumin, the most abundant circulating protein in plasma, constitutes a major oxygen radical scavenger and antioxidant defense against oxidants generated by both endogenous and exogenous substances (60, 61). This molecule exerts its effects through multiple binding sites and free radical scavenging properties (61). Previous studies utilizing free radical-induced hemolysis assays have demonstrated that over 70% of serum free radical scavenging activity is attributed to human serum albumin (62). Considering the potential of antioxidants to mitigate inflammatory reactions, the beneficial effects of albumin on cognitive function are biologically plausible. Additionally, given the reported inhibitory effect of albumin on the formation of amyloid-β peptide fibrils (63, 64), low albumin concentration may increase the risk of Alzheimer’s-type dementia. Therefore, from a perspective of Alzheimer’s disease prevention, clinicians should exercise greater vigilance to avoid a decrease in serum albumin levels, even within clinically normal ranges (65).

This study examined the backgrounds of patients with MMSE scores below 24, identifying characteristics such as advanced age, male sex, low hemoglobin levels, low albumin levels, and elevated serum creatinine levels. Evidence indicates that dementia is often inadequately diagnosed at the primary care stage. Among 146 patients not formally diagnosed with dementia, 72 individuals (49%) received a formal diagnosis after screening, with 69% categorized as “nonspecific cognitive impairment” (66). MCI is officially determined through comprehensive cognitive assessments by healthcare specialists, incorporating clinical examinations, medical histories, and often input from informants familiar with the patient. However, as this is not routinely conducted in primary care, there is a significant potential for delayed diagnosis. Cognitive impairment and MCI hold significant implications for patients and their families, necessitating primary care clinicians to adeptly identify and manage this prevalent disorder, especially as the elderly population continues to rise over the coming decades (67). MMSE possesses substantial evidence supporting its use and adequate testing accuracy, yet its utility is constrained by longer administration time (10–15 min). Therefore, there is a demand for tools that automatically estimate cognitive impairment risks using common information such as health checkup data including basic blood test data.

In this study, the correlation coefficient between the MMSE scores estimated by DLM based on blood test data obtained from EHR and the actual MMSE scores was at most 0.380, indicating a poorer predictive performance compared to our previous research results (r = 0.66) (22). Further refinement is essential for practical application due to the model’s limited accuracy (see the comment in the limitations below). Models based solely on EHR data may exhibit bias, as they lack crucial information about other social determinants of daily function and health (e.g., physical function, social connections), potentially restricting predictive performance (68). A review of 116 studies revealed that most utilized magnetic resonance imaging and positron emission tomography data (69). Generally, more complex models combining multi-modal and multi-dimensional data (neuroimaging, clinical, cognitive, genetic, behavioral), such as those based on deep learning, achieved the highest performance (69). Successful artificial intelligence systems require machine learning components to process structured data (images, electrophysiological data, genetic data) and natural language processing components to mine unstructured text (70). As reported, incorporating multi-modal data as input may further enhance predictive performance (71). However, acquiring such multi-modal data entails significant trade-offs. Magnetic resonance imaging and positron emission tomography involve high costs and limited measurement environments, potentially limiting widespread application among regionally residing elderly individuals. Needle insertion and the use of radioactive substances are further drawbacks of positron emission tomography (72). Moreover, in regions with limited medical resource availability, cognitive impairment screening and diagnosis may be delayed or underestimated. Therefore, it may not be suitable for MCI screening in communities or underdeveloped regions. In contrast, the approach of this study required solely the utilization of basic blood test data collected during health examination and routine clinical care. Utilizing existing blood test data in EHR for secondary purposes allows for the use of low-cost, easily accessible variables as input, enabling the DLM to estimate cognitive function in a short time. Once blood test results are confirmed in EHR, cognitive function prediction can be instantly calculated, allowing for real-time alerts. Thus, our MCI prediction approach using blood test data has the potential to be valuable for primary screening of numerous subjects at the community level in a short timeframe. However, given the existing limitations in predictive performance, further research is needed to enhance its efficacy.

This study has several limitations. Firstly, due to the cross-sectional study design, causal relationships cannot be inferred. While low albumin levels may potentially lead to cognitive impairment, a reverse causation is also plausible, where cognitive impairment could result in malnutrition and subsequent albumin reduction (73). Establishing causation requires longitudinal studies. However, the primary objective of this study is not to demonstrate causation but to develop a high-throughput tool for primary screening of cognitive impairment in a community setting. Secondly, there are challenges in generalizing the results of this study. The prevalence of MCI is 10–20% among the elderly (7, 8). In contrast, in this study, 51.1% of individuals aged 65 or older were considered to have MCI with MMSE<24, which is a high proportion. This is influenced by the fact that the subjects of this study are patients who underwent MMSE at the hospital, i.e., those who experienced episodes where the physician deemed cognitive function examination necessary. Therefore, the study subjects may not represent a population reflective of the Japanese demographic, and the generalizability to a community-based elderly population remains uncertain. Thirdly, the low correlation coefficient between MMSE scores estimated from blood data and the actual MMSE scores. The MMSE scores estimated by DLM are based on blood test data reflecting systemic metabolic disorders and are not indicative of actual cognitive function (MMSE). This suggests that the cases in our study showed minimal impact of systemic metabolic disorders on cognitive function. For cases with no systemic metabolic issues but only brain-related problems (such as post-subarachnoid hemorrhage), the actual MMSE scores were lower than the estimated MMSE scores (22). Also, when DLM trained with cases of elderly patients with severe arteriosclerosis was applied to relatively younger patients without advanced arteriosclerosis, the estimated MMSE scores were lower than the actual MMSE scores (22). These results suggest the need to combine other machine learning methods that directly reflect brain function (such as facial (20), vocal (21)) with the current approach. Additionally, they indicate the necessity of using multiple DLMs trained with teacher groups of patients with various pathologies for evaluation.

## 5 Conclusion

An analysis of blood test data from 1,352 patients, coupled with MMSE, was conducted to examine the relationship between albumin and cognitive function. Additionally, a machine learning model was constructed for estimating cognitive function. Statistical analysis revealed a significant association between low albumin levels and impaired cognitive function in individuals aged 65 and above. Employing both conventional machine learning algorithms and DLM, we constructed a predictive model for MMSE scores, with the DLM demonstrating the optimal result. Especially for those aged 65 or above, the suggestion was that, in addition to age, albumin serves as a significant predictive factor for estimating cognitive function.

## Data availability statement

The datasets presented in this article are not readily available because of ethical reasons. Requests to access the datasets should be directed to corresponding author.

## Ethics statement

The studies involving humans were approved by the ethics committee of Osaka Medical and Pharmaceutical University. The studies were conducted in accordance with the local legislation and institutional requirements. Written informed consent for participation was not required from the participants or the participants’ legal guardians/next of kin since this is a retrospective observational study without intervention or invasion.

## Author contributions

KK: Data curation, Formal analysis, Investigation, Methodology, Software, Validation, Visualization, Writing – original draft. TH: Data curation, Formal analysis, Investigation, Methodology, Resources, Software, Validation, Visualization, Writing – original draft, Writing – review & editing. AI: Validation, Writing – review & editing. KO: Validation, Writing – review & editing. EU: Validation, Writing – review & editing. KS: Conceptualization, Funding acquisition, Project administration, Supervision, Validation, Writing – review & editing.
