# Supplementary material for: Importance of serum albumin in machine learning-based prediction of cognitive function in the elderly using a basic blood test
Source: Front Neurol. 2024 Jul 24;15:1362560. doi: 10.3389/fneur.2024.1362560 (PMC11303288; doi:10.3389/fneur.2024.1362560)
Supplement: Supplementary file 1 [file Table_1.docx]

Supplementary material

Table S1 Background of the excluded patients

| Backgrounds | Overall |
| --- | --- |
| Patients, n (%) | 3665 (100) |
| MMSE, median (IQR) | 24 (21–27) |
| MMSE, n (%) |  |
| <24 | 1705 (46.5) |
| ≥24 | 1960 (53.5) |
| Age, year, median (IQR) | 78 (72–82) |
| Age, n (%) |  |
| <65 | 376 (10.3) |
| ≥65 | 3289 (89.7) |
| Sex, n (%) |  |
| Female | 1970 (53.8) |
| Male | 1695 (46.2) |

IQR: interquartile range, MMSE: Mini Mental State Examination

Table S2 Relationship between MMSE and patient background

| Backgrounds | Overall | MMSE<24 | MMSE≥24 | *p*-value |
| --- | --- | --- | --- | --- |
| Patients, n (%) | 1352 (100) | 653 (48.3) | 699 (51.7) |  |
| MMSE, median (IQR) | 24 (22–27) | 22 (21–22) | 27 (26–28) | <0.001^*^ |
| MMSE, n (%) |  |  |  |  |
| <24 |  |  |  |  |
| ≥24 |  |  |  |  |
| Age, year, median (IQR) | 77 (72–82) | 79 (74–83) | 75 (70–80) | <0.001^*^ |
| Sex, n (%) |  |  |  | 0.002^*^ |
| Female | 717 (53.0) | 317 (48.5) | 400 (57.2) |  |
| Male | 635 (47.0) | 336 (51.5) | 299 (42.8) |  |
| WBC, 10^3^/µL, median (IQR) | 5.78 (4.77–7.11) | 5.91 (4.81–7.30) | 5.66 (4.73–6.97) | 0.018^*^ |
| RBC, 10^6^/µL, median (IQR) | 4.06 (3.68–4.42) | 3.97 (3.52–4.37) | 4.12 (3.77–4.47) | <0.001^*^ |
| Hemoglobin, g/dL, median (IQR) | 12.7 (11.5–13.7) | 12.4 (10.9–13.5) | 12.8 (11.8–13.7) | <0.001^*^ |
| Hematocrit, %, median (IQR) | 38.2 (34.9–41.2) | 37.7 (33.3–40.9) | 38.6 (35.8–41.5) | <0.001^*^ |
| MCV, fL, median (IQR) | 94.2 (90.8–98.1) | 94.5 (91.0–98.6) | 94 (90.6–97.5) | 0.020^*^ |
| MCH, pg, median (IQR) | 31.2 (30.1–32.4) | 31.3 (30.0–32.6) | 31.2 (30.2–32.4) | 0.506 |
| MCHC, %, median (IQR) | 33 (32.2–33.7) | 32.9 (32.2–33.6) | 33.1 (32.4–33.8) | 0.003^*^ |
| Platelets, 10^3^/µL, median (IQR) | 210 (170–255) | 207 (168–259) | 211 (173–252) | 0.881 |
| Total protein, g/dL, median (IQR) | 6.8 (6.4–7.2) | 6.8 (6.3–7.2) | 6.9 (6.5–7.2) | <0.001^*^ |
| Albumin, g/dL, median (IQR) | 3.9 (3.5–4.2) | 3.7 (3.3–4.1) | 4 (3.7–4.2) | <0.001^*^ |
| Albumin-globulin ratio, median (IQR) | 1.3 (1.1–1.5) | 1.2 (1.0–1.4) | 1.4 (1.2–1.5) | <0.001^*^ |
| AST, U/L, median (IQR) | 21 (17–26) | 20 (17–26) | 21 (17–26) | 0.547 |
| ALT, U/L, median (IQR) | 16 (11–23) | 15 (11–23) | 17 (12–23) | 0.005^*^ |
| γ-GTP, U/L, median (IQR) | 23 (16–39) | 23 (16–39) | 23 (16–40) | 0.957 |
| Total cholesterol, mg/dL, median (IQR) | 188 (162–216) | 186 (160–216) | 191 (166–217) | 0.039^*^ |
| Triglyceride, mg/dL, median (IQR) | 105 (76–146) | 104 (76–142) | 108 (77–149) | 0.191 |
| BUN, mg/dL, median (IQR) | 17 (13–21) | 17 (13–22) | 16 (13–20) | 0.002^*^ |
| Creatinine, mg/dL, median (IQR) | 0.81 (0.66–1.01) | 0.84 (0.68–1.07) | 0.79 (0.65–0.96) | <0.001^*^ |
| Uric acid, mg/dL, median (IQR) | 4.9 (4.0–6.0) | 4.9 (3.9–6.0) | 4.9 (4.0–5.9) | 0.902 |
| Glucose, mg/dL, median (IQR) | 109 (94–147) | 111 (94–147) | 109 (94–146) | 0.720 |
| Sodium, mEq/L, median (IQR) | 141 (139–143) | 141 (139–143) | 141 (139–143) | 0.282 |
| Potassium, mEq/L, median (IQR) | 4.2 (3.9–4.5) | 4.1 (3.9–4.4) | 4.2 (3.9–4.5) | 0.050 |
| Chloride, mEq/L, median (IQR) | 104 (102–106) | 104 (102–106) | 104 (102–106) | 0.716 |

ALT: alanine aminotransferase, AST: aspartate aminotransferase, BUN: blood urea nitrogen, γ-GTP: γ-glutamyl transpeptidase, IQR: interquartile range, MCH: mean corpuscular hemoglobin, MCHC: mean corpuscular hemoglobin concentration, MCV: mean corpuscular volume, MMSE: Mini Mental State Examination, RBC: red blood cell, WBC: white blood cell

^*^: *p*<0.05
